# Molecular Characteristics and Pathogenicity of Porcine Reproductive and Respiratory Syndrome Virus (PRRSV) 1 in Taiwan during 2019–2020

**DOI:** 10.3390/life13030843

**Published:** 2023-03-21

**Authors:** Fu-Chun Hsueh, Kun-Lin Kuo, Feng-Yang Hsu, Sheng-Yuan Wang, Hsien-Jen Chiu, Meng-Tien Wu, Chuen-Fu Lin, Yu-Han Huang, Ming-Tang Chiou, Chao-Nan Lin

**Affiliations:** 1 Animal Disease Diagnostic Center, College of Veterinary Medicine, National Pingtung University of Science and Technology, Pingtung 91201, Taiwan; 2 Department of Veterinary Medicine, College of Veterinary Medicine, National Pingtung University of Science and Technology, Pingtung 91201, Taiwan

**Keywords:** PRRSV 1, phylogenetic analysis, challenge experiment, Taiwan

## Abstract

Two variants of porcine reproductive and respiratory syndrome virus (PRRSV), PRRSV 1 and PRRSV 2, have caused abortion in pregnant sows and respiratory distress in nursery pigs worldwide. PRRSV 2 has been thoroughly researched in Taiwan since 1993; however, the first case of PRRSV 1 was not reported until late 2018. To decipher the genetic characteristics of PRRSV 1 in Taiwan, open reading frame 5 (ORF5) genes of PRRSV 1 strains collected from 11 individual pig farms in 2019–2020 were successfully sequenced. All Taiwanese ORF5 sequences were closely related to Spanish-like PRRSV strains, which are considered to share a common evolutionary origin with the strain used for the PRRSV 1 vaccine. Analyses of amino acid (aa) and non-synonymous substitutions showed that genetic variations resulted in numerously specific codon mutations scattered across the neutralizing epitopes within the ORF5 gene. The PRRSV 1 challenge experiment disclosed the pathogenetic capability of the NPUST2789 isolate in nursery pigs. These findings provide comprehensive knowledge of the molecular diversity of the PRRSV 1 variant in local Taiwanese fields and facilitate the development of suitable immunization programs against this disease.

## 1. Introduction

Since porcine reproductive and respiratory syndrome virus (PRRSV) was first discovered in Europe and the USA [1,2], it has resulted in middle- or late-term reproductive failure in pregnant sows and respiratory disorders in nursery pigs. This porcine disease, called porcine reproductive and respiratory syndrome (PRRS), also devastates the swine industry, causing great economic losses worldwide [3]. As an enveloped, positive-stranded RNA virus, PRRSV belongs to the order *Nidovirales*, family *Arteriviridae*, genus *Betaarterivirus* and can be classified into two species, *Betaarterivirus suid 1* (PRRSV 1, formerly called the European type) and *Betaarterivirus suid 2* (PRRSV 2, formerly called the North American type), with approximately 44% genetic differences between each other [4,5]. Both PRRSV strains manifest the same clinical signs in naïve sows and gilts [6]. In Taiwan, the first outbreak of PRRSV 2 was reported in 1993 [7]. Since then, PRRSV 2 has uninterruptedly circulated in swine herds in the absence of effective management or strategies against it. Later, PRRSV 1 emerged in Taiwanese fields in late 2018, hindering the birth of piglets and undermining the confidence in vaccination [8].

Porcine reproductive and respiratory syndrome virus is approximately 15.1 kb in length and encodes 10 open reading frames (ORFs), ORF1a, 1b, 2a, 2b, 3, 4, 5, 5a, 6 and 7 [9]. Among them, the highly mutated ORF5 gene, which encodes glycoprotein 5 (GP5), plays a pivotal role in inducing viral neutralizing antibodies and imparting cross-protection to pigs against heterologous PRRSV strains [10,11]. GP5 consists of three regions, the ectodomain, the hydrophobic region and the putative endodomain [12]. Specifically, two neutralizing epitopes have been identified within the ectodomain, the N-terminal ectodomain (amino acid (aa) 29–35) [12] and the ectodomain epitope (aa 38–54) [13], which are recognized by neutralizing monoclonal antibodies. Additionally, an immunodominant T-epitope, also named the major histocompatibility complex class I (MHC-I) epitope, has been located in the region of aa 117–128. This epitope has the capability of activating cell-mediated immunity against PRRSV by stimulating IFN-γ secreting cells [14]. On the other hand, a deceptive “decoy epitope” situated in the hypervariable area of GP5 plays an important role in evading neutralizing responses by the N-glycan-shielding mechanism [15,16]. The variation in N-glycosylation sites may interfere with the recognition by neutralizing antibodies and further compromise the immunogenicity of neutralizing epitopes since specific glycosylation sites could differ during viral replication in vivo [17,18].

As a highly mutated virus, PRRSV 1 was initially separated into four different genotypes based on the ORF5 sequence [19]. A few years later, the novel phylogenetic classification system further divided PRRSV 1 into 12 sub-clades to explicitly accentuate the genomic diversity [20]. Following the incorporation of nucleotide sequences from central and eastern European countries, a more solid classification system proposed that PRRSV 1 should be categorized into three lineages [21]. Among them, Lineage 1 is composed of the prototype PRRSV 1, Lelystad strain as well as PRRSV DV (Merck, Sharp and Dohme Animal Health, Rahway, NJ, USA), Pyrsvac-183 (Laboratorios Syva, S.A., León, Spain), Amervac PRRSV (Laboratorios Hipra, S.A., Girona, Spain) and Unistrain PRRSV (Laboratorios Hipra, S.A., Girona, Spain) vaccine strains [21]. Currently, live-attenuated or modified live vaccines have similar concerns over safety, meager heterologous cross-protection and the reversion of virulence [22,23]. This is the common warning of all commercial PRRSV modified live vaccines, which emphasizes the importance of adjustable immune programs. Thus, the elucidation of the molecular diversity of PRRSV 1 has become an essential task to illuminate the actual epidemic in the field and further facilitate more appropriate vaccination strategies for disease control. Here, this study records the molecular characteristics of the ORF5 gene and pathogenicity of PRRSV 1 in Taiwan following the first outbreak in 2018. These results provide the in situ status of currently circulating PRRSV 1 and will promote the advancement of effective procedures to deal with this virus.

## 2. Materials and Methods

### 2.1. Sample Collection

In 2019–2020, clinical specimens (serum or tissues) were collected from 15,034 pigs for PRRSV detection from northern, central, southern and eastern Taiwan. Severe reproductive and/or respiratory epidemics took place on 11 different pig farms in central and southern Taiwan. On those farms, pregnant sows manifested abortion, prematurity and stillbirth, and nursery pigs had high fever, severe dyspnea and inappetence accompanied by edematous eyelids and discolored ears. Detailed information on vaccination and background history is summarized in Table 1. Blood samples and lung tissues were submitted to the Animal Disease Diagnostic Center at National Pingtung University of Science and Technology for molecular diagnosis. All specimens were confirmed as PRRSV 1-positive by quantitative reverse transcription polymerase chain reaction (RT-qPCR) performed as previously mentioned [8].

### 2.2. Amplification of the ORF5 Gene and Phylogenetic Analysis

Viral nucleic acids were first extracted using the MagNA Pure LC total nucleic acid isolation kit (Roche Diagnostics, Mannheim, Germany) and then used to synthesize complementary DNA (cDNA) using the PrimeScript^TM^ RT reagent kit (Takara Bio Inc., Kusatsu, Shiga, Japan). Two microliters of template cDNA was amplified utilizing KAPA HiFi HotStart ReadyMix (Roche), and two primer pairs were designed outside of the ORF5 gene (Table 2) under the following thermal conditions: initial denaturation at 95 °C for 3 min; 40 cycles of 98 °C for 20 s, 56 °C for 30 s and 72 °C for 50 s; and a final extension at 72 °C for 50 s. PCR products were subjected to 2% agarose gel electrophoresis, visualized by ultraviolet illumination after ethidium bromide staining, and the TA cloning of ORF5 gene as described in a previous study [8] was performed and confirmed by nucleotide sequencing (Mission Biotech Inc., Taipei, Taiwan). Multiple sequence alignment of historical global PRRSV 1 reference strains was established using Clustal W in the Molecular Evolutionary Genetics Analysis (MEGA), version 11 software program to construct the phylogenetic trees by the maximum likelihood estimation (MLE) method in the Kimura 2-parameter (K2P) mode using a discrete gamma distribution and bootstrap methods estimated for 1000 replications.

### 2.3. Identification of Selected Sites of the ORF5 Gene

To precisely deduce the positive/diversifying and negative/purifying selection sites, four algorithms, Fixed Effects Likelihood (FEL), Single-Likelihood Ancestor Counting (SLAC), Fast Unconstrained Bayesian AppRoximation (FUBAR) and Mixed Effects Model of Evolution (MEME), were performed. The ratio of non-synonymous substitutions (beta) and synonymous substitutions (alpha) per site based on the maximum likelihood tree was calculated using the FEL, SLAC and MEME methods at a significance level of 0.05 [24], while the posterior probability of positive or negative selection at a single site was evaluated using the FUBAR method with a threshold set at 0.95 [25]. All of the abovementioned methods were implemented on the Datamonkey web server of the HyPhy software package (http://www.datamonkey.org (accessed on 14 February 2022)) [26].

### 2.4. Animal Experiment

To evaluate the pathogenicity of the PRRSV 1 strain in pigs, we performed a previous isolate, NPUST2789 (GeneBank accession number: MN242825) [8] as the PRRSV 1 challenged variant in this experiment. A total of 21 4-week-old Landrace × Yorkshire × Duroc crossed-breed pigs were automatically separated into five groups: the high-inoculated (HIN) group (*n* = 3), the high-inoculated contacted (HC) group (*n* = 6), the low-inoculated (LIN) group (*n* = 3), the low-inoculated contacted (LC) group (*n* = 6) and the vehicle group (*n* = 3). No pigs received the PRRSV vaccines in this study. All pigs were first confirmed to be PRRSV 1-seronegative negative and then intranasally challenged with 2 mL of 10^5^ and 10^3^ TCID_50_/mL NPUST2789 in the HIN and LIN group, respectively. As for the HC and LC groups, those pigs were herded in the same batch as pigs from the HIN and LIN groups without being challenged. Body weights and body temperatures of pigs were monitored weekly and daily, respectively. Serum samples from pigs were collected in Vacutainer^®^ Plus Plastic SSTTM tubes with polymer gel (BD Medical, East Rutherford, NJ, USA) at 0-, 3-, 7-, 10- and 14-days post-inoculation (DPI) for evaluating both PRRSV 1 antibodies and viral loads by using the IDEXX PRRS X3 Ab Test kit (IDEXX, Westbrook, ME, USA) and RT-qPCR, respectively. After euthanasia for all PRRSV 1-challenged pigs, all lobes of the lung were excised, fixed in 10% neutral-buffered formalin, paraffin-embedded and stained with hematoxylin for examining the PRRSV 1 invasion in lung tissue sections.

### 2.5. Statistics

Body weights, body temperatures and viral loads were depicted and statistically measured using GraphPad Prism 9.5.0 (GraphPad Software, San Diego, CA, USA) with two-way analysis of variance (ANOVA). Statistically significant differences were represented by *p*-values < 0.001.

## 3. Results

### 3.1. PRRSV 1 Nucleotide Sequencing and Phylogenetic Analysis of the ORF5 Gene

**Figure 1:**
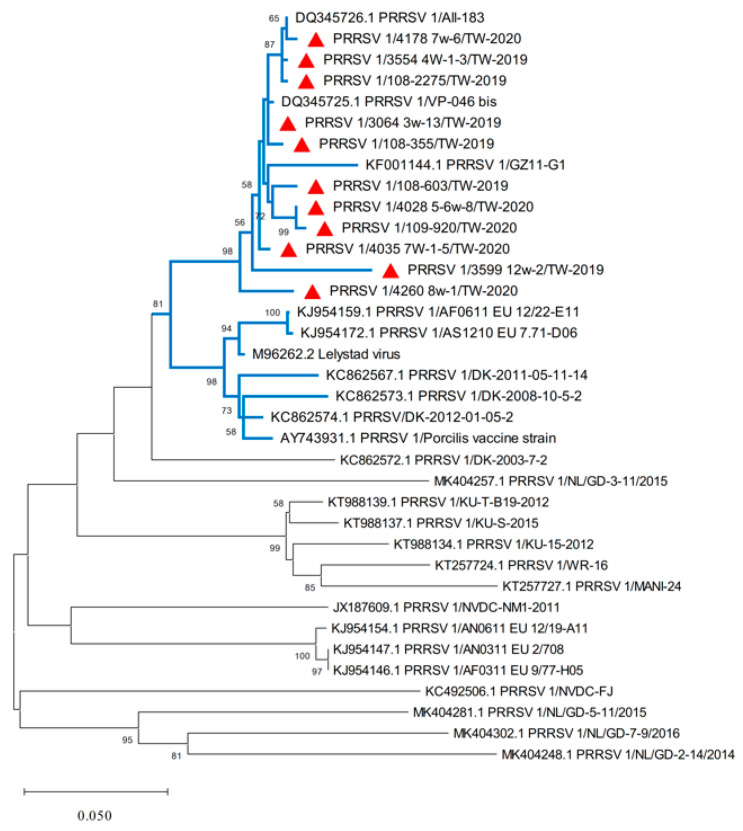
A phylogenetic tree based on the ORF5 gene of PRRSV 1 sequences was constructed by maximum likelihood estimation (MLE) using the Kimura 2-parameter (K2P) mode under the gamma distribution with 1000 bootstrapping replicates. Horizontal branch lengths indicate genetic distances among different viral strains. The red triangle represents the Taiwan PRRSV 1 strains isolated in 2019–2020 within the blue cluster. The scale bar indicates nucleotide substitutions per site.

A total of 5464 (36.3%) samples from 15,034 pigs were positive for PRRSV. Among them, 158 (2.9%) pigs were PRRSV 1-positive, 5275 (96.5%) were PRRSV 2-positive, and 31 (0.6%) were positive for both PRRSV 1 and 2 (Table 3). Severe reproductive and/or respiratory epidemics that took place on 11 different pig farms were further analyzed in the present study. A total of 11 full-length ORF5 genes were successfully amplified and sequenced. The location of the herds encompassed five separate counties, Pingtung County (four herds), Kaohsiung City (two herds), Miaoli County (one herd), Yunlin County (three herds) and Hsinchu County (one herd), with details on case history and vaccination information listed in Table 1. The nucleotide lengths of the ORF5 gene of the PRRSV 1 strains in this study were all 606 bp. The ORF5 nucleotide sequences of these 11 strains (GenBank accession no. OM677752-OM6777762) showed 93.7–99.7% identity to each other. Compared with the rest of the PRRSV 1 strains in the blue cluster and the remaining unmarked viral variants (Figure 1), our Taiwan PRRSV 1 strains manifested 87.9–99.8% and 79.9–88.2% nucleotide identities, respectively. Phylogenetic analysis of all Taiwanese PRRSV 1s (Figure 1) revealed that they belonged to Lineage 1 based on the previous classification system [21], which was established from prototype PRRSV isolates in Western Europe in 1991. These local variants were closely related to the PRRSV 1 strains Vp-046_bis and All-183 (GenBank accession nos. DQ345725 and DQ345726), with nucleotide identities of 99.84% and 99.67%, respectively.

### 3.2. Amino Acid Analysis of ORF5 of PRRSV 1

**Figure 2:**
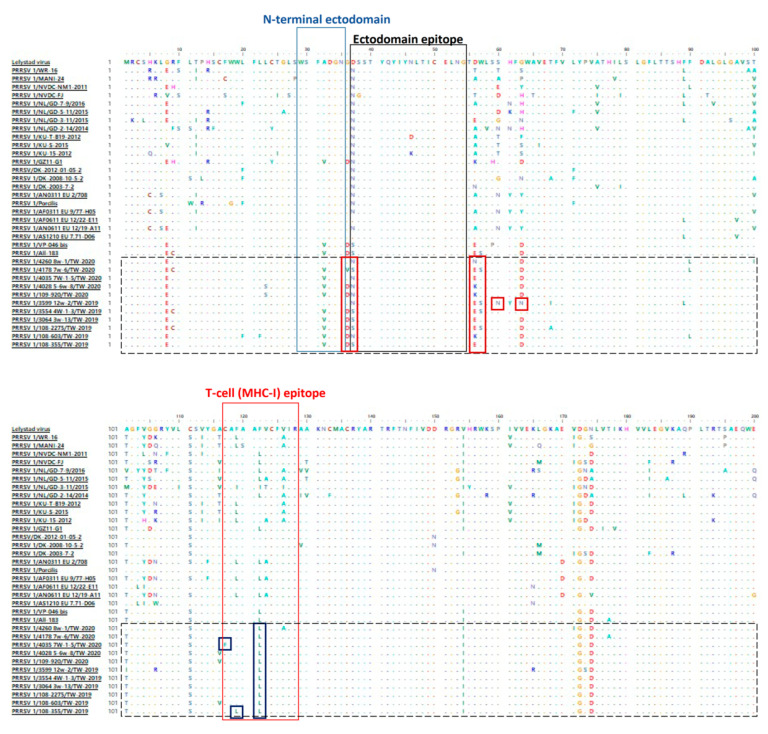
Alignment of amino acid sequences of glycoprotein 5 of 11 Taiwanese PRRSV 1 variants along with historical global viral strains. Potential neutralizing epitopes are marked above. Ellipses represent the amino acids identical to the reference sequence.

Multiple amino acid alignment of ORF5 showed that all 11 Taiwanese PRRSV isolates in 2019–2020 displayed prolific posterity aa sites analogous to the Spanish-like PRRSV 1 strains [27]. However, the PRRSV 1 variants in Taiwan still exhibited numerous aa mutations at a percentage of 4.6–20.3% in comparison with all the other historical global viral strains. These aa mutations scattered across neutralizing epitopes of the ORF5 gene comprised A^32^V in the N-terminal ectodomain, D^37^N/S in the ectodomain and C^117^F, F^119^L and F^122^L in the T-cell epitope (Figure 2). Furthermore, some unique aa mutations were also identified in the majority of Taiwanese PRRSV strains, including G^8^E, A^32^V, G^36^D/V, D^56^E/K, G^63^D, A^101^T/I, C^111^S, V^154^I, D^172^G and N^174^D (Figure 2). Of note, 3 aa mutations were discovered at the N-glycosylation site, 2 of which, S^60^N and G^63^N, were discovered in the PRRSV 1/3599 12w-2/TW-2019 strain, and 1 of them, D^56^N, was noticed in the PRRSV 1/4260 8w-1/TW-2020 variant (Figure 2).

### 3.3. Estimation of Non-Synonymous and Synonymous Substitutions of the ORF5 Gene

**Figure 3:**
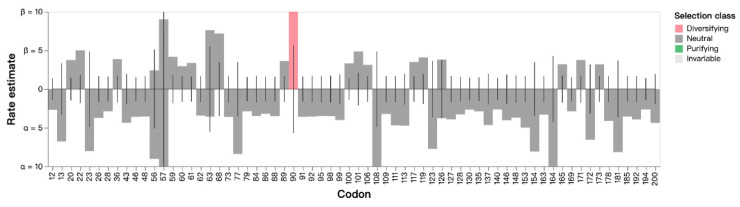
Maximum likelihood estimates of non-synonymous rates (beta; β) and synonymous rates (alpha; α) at each site are shown as bars. The line shows the estimates under the null model (α = β). Estimates above 10 at codon 90 are censored at the site under diversifying positive selection at *p* < 0.05.

Non-synonymous (beta) and synonymous (alpha) estimates of each codon among 11 Taiwanese variants and 6 historical variants, including Vp-046_bis, All-183, DV, Lelystad virus, AF0611 and AS1210 (Figure 1), were analyzed. The average value of beta/alpha was 0.287. The results of FEL analysis indicated that 1 specific site at codon 90 was identified under diversifying positive selection (Figure 3) with a likelihood ratio test (LRT) at 5.063 and *p* value = 0.024. Using the FUBAR method, a similar result was obtained at codon 90 by positive selection, but 5 codon sites were also highlighted by negative selection (Table 4). The MEME method indicated that a distinct site at codon 36 was computed by positive selection with a non-synonymous substitution rate of 220.51, an LRT of 5.78, and a *p* value of 0.03. No positive or negative selection was found by the SLAC method.

### 3.4. Pathogenicity of Virulent NPUST2789 Challenge in Nursery Pigs

**Figure 4:**
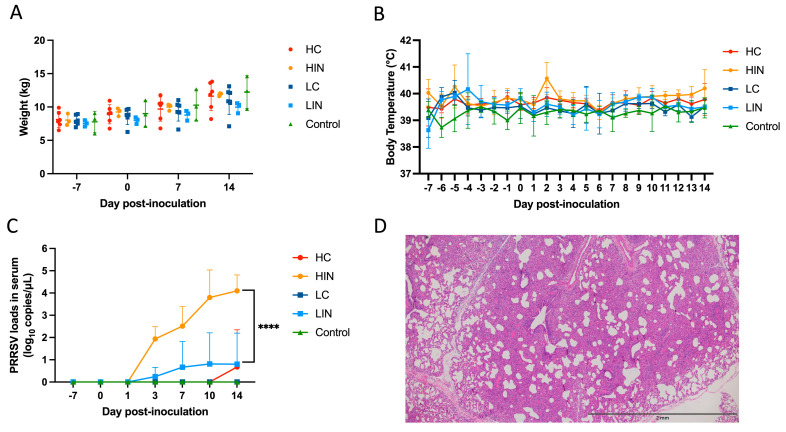
Body weights (**A**), body temperature (**B**), PRRSV loads in serum (**C**) and PRRSV-challenged lesions suggestive of diffuse and severe interstitial pneumonia (**D**) in pigs following the NPUST2789 challenge. The PRRS viral load was detected by a probe−based quantitative reverse transcription PCR (RT−qPCR) and transformed into the log_10_ copies/µL. The error bars indicate the standard deviation. Statistically significant differences are marked as **** (*p* < 0.001).

The variations of both body weights and body temperatures in each group are recorded in Figure 4A,B. No statistically significant difference in weekly body weights and daily body temperatures was observed among different groups. During this study, no sign of fever was discovered in a single pig. Regarding the PRRSV antibodies, all pigs in the HIN group and 1 pig in the LIN group showed positive after 3 DPI. The levels of PRRSV 1 viral loads in each group were depicted in Figure 4C. In the HIN group, the average viral titers in serum were 1.94 ± 0.45 log_10_ copies/µL at 3 DPI, and gradually climbing at 7, 10 and 14 DPI with viral titers of 2.51 ± 0.72, 3.80 ± 1.01 and 4.10 ± 0.58 log_10_ copies/µL, respectively. As to the LIN group, only 1 pig showed positive viral titers starting from 0.24 ± 0.34 log_10_ copies/µL at 3 DPI, 0.67 ± 0.94 log_10_ copies/µL at 7DPI, to a viral peak of 0.81 ± 1.14 log_10_ copies/µL at 10 DPI and finally 0.81 ± 1.14 log_10_ copies/µL at 14 DPI. In the HC group, the viral titer of 0.80 ± 1.13 log_10_ copies/µL was discovered at 14 DPI. Significantly statistical differences (*p* < 0.001) were noticed at 14 DPI. Moreover, the histopathological examination manifested that the PRRSV-associated lesions of severe interstitial pneumonia were only noted in pigs at the HIN group (Figure 4D).

## 4. Discussion

The earliest case of field-circulating PRRSV 1 in Taiwan was discovered in 2018, which caused numerous abortions and stillbirths in sows [8]. Thereafter, no study focused on the phylogenetic variation of PRRSV 1 in Taiwan. To fill this gap, 11 full-length ORF5 genes of PRRSV 1 were sequenced from 11 separate pig farms across 5 different geographic areas in Taiwan. All Taiwanese strains were closely related to historic Spanish PRRSV 1s, Vp-046_bis and All-183, which had been designated Amervac PRRSV (Laboratorios Hipra) and Pyrsvac-183 (Laboratorios Syva), respectively. Multitudinous amino acid mutations scattered across the neutralizing epitopes of Taiwanese PRRSV 1 glycoprotein 5 were identified and compared with each other or with historical global viral strains. In terms of sample sizes, this limitation of only 11 sequences is due the low detection rate of PRRSV 1 (2.9%) during 2019–2020 in Taiwan. To our knowledge, this is the first study to demonstrate the phylogenetic characteristics of PRRSV 1 in Taiwan. These findings can serve as essential information for developing a useful strategy for disease control.

Porcine reproductive and respiratory syndrome virus 1 has been recognized as a pathogen with high evolutionary rates, especially in the ORF5 gene [28], which resulted in recombinant or mutation events [29]. Among the ectodomain and the hydrophobic region, Taiwanese PRRSV 1 variants exhibited numerous aa mutations scattered across the N-terminal domain (1 site), the ectodomain epitope (1 site) and the rest of the region (14 sites). Of note, unique aa mutations at specific N-glycosylation sites, such as D^37^N, D^56^N, S^60^N and G^63^N, are closely associated with immune evasion, inhibition of viral recognition by the host and loss of neutralizing capabilities [27,30]. Another aa mutation (R^9^C) at a conserved cysteine residue, which might alter the folding and function of glycoprotein [31], was noticed in three of our sequences. Furthermore, amid the T-cell epitope, four aa mutations, including C^117^F at the conserved cysteine site and F^119^L, F^122^L and V^126^A, have also been discovered in vaccine-derived wild-type PRRSV 1 [31,32]. Collectively, these results might be a feasible explanation for the incompletion of PRRSV vaccines; in addition, the highest titer of PRRS 1 RNA was found in the diseased pigs (Table 1). Therefore, challenge trials of local wild-type PRRSV 1 isolate are crucial to demonstrate its actual pathogenicity. In this study, we evaluated the previous PRRSV 1 isolate, NPUST2789, challenged in nursery pigs and verified the PRRSV 1 viral loads in serum and the PRRSV 1-related lesion in the lung tissue sections, which point outs that Taiwanese wild-type PRRSV 1s have potentially high infectious capabilities.

The primary concern of live-attenuated vaccines is the recovery of virulence [23]. In Asia, cases of vaccine-derived wild-type PRRSV 1 infection are not infrequent [29,33]. One Thai study documented that most of their isolates shared over 99% identities in the ORF5 gene with the European vaccine, even though modified live virus (MLV) vaccines for PRRSV 1 have not been used in local fields [33]. Interestingly, there were comparable events on Farms E and K, which did not receive the PRRSV 1 vaccine. This might be due to the international trade of breeding pigs or semen from foreign countries and the close interactions between farms that have administered the live-attenuated PRRSV 1 vaccine. In Korea, two studies demonstrated that the Korean viral strains were derived from a common ancestor of the Spanish type 1 PRRSV [32] and the VP-046-like viral strain [34]. A similar study in China also indicated that their local isolates might have originated from Amervac PRRSV vaccine-related isolates and undergone further genetic evolution [35]. In European countries, two studies in Denmark [31] and the Netherlands [36] mentioned that both their wild-type isolates strongly resembled the PRRSV vaccine strain DV. In the current study, our PRRSV 1 variants were comparable to the Spanish-like PRRSV 1 vaccine strain. Furthermore, potential recombinant PRRSV strains derived from vaccines and wild-type viral strains had also discovered what was mentioned above. PRRSV MLV vaccines do confer late but effective protection against homologous wild-type virus, while only limited protection or no protection against heterologous strains [37]. Therefore, both efficacy and safety should be concerns for the use of MLV vaccines [38]. Taken together, authorized vaccines are still the optimal solution against PRRSV, but they must be administered only under the proper evaluation by veterinarians, and the vaccine schedule must be adjusted for adequate immunization. Moreover vaccination and strict external biosecurity protocols are still the only choice for the control and prevention of PRRSV in Taiwan in the future.

## 5. Conclusions

In the present study, we provide up-to-date knowledge with regard to the phylogenetic diversity of circulating PRRSV 1 in Taiwan after the first outbreak in late 2018. Miscellaneous amino acid mutations in the glycoprotein 5 might be ascribed to the significant evolutionary capabilities of PRRSV 1. Moreover, the challenge experiment proved that the PRRSV 1 isolate, NPUST2789, has the ability to induce viremia and to damage lungs in nursery pigs, and accordingly, this highlights the urgency of the appropriate immunization program using live-attenuated vaccines. Our results can serve as the basis for developing suitable strategies for PRRS control.

## Figures and Tables

**Table 1:** Complete information of PRRSV 1 in each pig farm.

Farm	Collection Time	Location	Scale of Herd	Type of Vaccine ^1^	Age in Weeks	Animal Condition ^2^	Quantitation of PRRSV 1/PRRSV 2 Loads ^3^	Sample
Farm A	January 2019	Pingtung	1400 sows	Live attenuated PRRSV 1	3	Sick	1.10 × 10^6^/− ^4^	NPUST3064
Farm B	March 2019	Pingtung	170 sows	Live attenuated PRRSV 1	8	Sick	5.85 × 10^6^/−	NPUST108-355
Farm C	April 2019	Kaohsiung	200 sows	Live attenuated PRRSV 1	6–7	Sick	1.87 × 10^6^/−	NPUST108-603
Farm D	August 2019	Kaohsiung	140 sows	Live attenuated PRRSV 1 and Modified live PRRSV 2	4	Normal	1.90 × 10^4^/−	NPUST3554
Farm E	August 2019	Pingtung	480 sows	Modified live PRRSV 2	12	Normal	4.40 × 10^4^/−	NPUST3599
Farm F	November 2019	Miaoli	400 sows	Live attenuated PRRSV 1	5	Sick	1.77 × 10^6^/−	NPUST108-2275
Farm G	February 2020	Yunlin	900 sows	Live attenuated PRRSV 1	5–6	Sick	2.26 × 10^5^/−	NPUST4028
Farm H	February 2020	Pingtung	250 sows	Live attenuated PRRSV 1	7	Normal	7.1 × 10^4^/5.2 × 10^3^	NPUST4035
Farm I	March 2020	Yunlin	200 sows	Live attenuated PRRSV 1	7	Normal	1.7 × 10^4^/−	NPUST4178
Farm J	March 2020	Hsinchu	200 sows	Non provided	8	Normal	4.7 × 10^4^/−	NPUST4260
Farm K	April 2020	Yunlin	1100 hogs	Modified live PRRSV 2	5	Sick	6.64 × 10^7^/−	NPUST109-920

^1^ Vaccination program of the PRRSV vaccine: every 3 months for sows and at 7–10 days of age for piglets; ^2^ definition of animal condition: “Sick” means that PRRS-related signs were obviously noticed in pigs of the farm; ^3^ units of PRRSV loads: copies/µL. ^4^—means PRRSV qPCR negative.

**Table 2:** The list of primers used for sequencing in this study.

Name of Primers	Sequences
EU-ORF5-F1	5′-TGCATTTCYTGACACCATC-3′
EU-ORF5-R1	5′-CCCBARRAGTCGGCCRCGWGA-3′
EU-ORF5-F2	5′-ATCYRCAATGAGGTGGGCTAC-3′
EU-ORF5-R2	5′-ACYTTNAGGGCRTADATCAT-3′

**Table 3:** Positive rates of PRRSV 1 and PRRSV2 during 2019–2020.

Year	Number	Positive (%)	PRRSV 1 Only (%)	PRRSV 2 Only (%)	PRRSV 1 + PRRSV 2 (%)
2019	8852	2864 (32.4)	108 (3.8)	2747 (95.9)	9 (0.3)
2020	6182	2600 (42.1)	50 (1.9)	2528 (97.2)	22 (0.9)
Total	15,034	5464 (36.3)	158 (2.9)	5275 (96.5)	31 (0.6)

**Table 4:** Results of positive and negative selection by the FUBAR method.

Codon Site	Mean Posterior Beta-Alpha	Negative Selection	Positive Selection	
		Prob [Alpha > Beta] ^a^	Prob [Alpha < Beta] ^b^	Bayes Factor [Alpha < Beta] ^c^
77	−12.955	**0.974**	0.017	0.023
90	9.578	0.018	**0.961**	**31.945**
108	−17.870	**0.984**	0.009	0.012
123	−14.816	**0.979**	0.014	0.018
164	−20.986	**0.988**	0.007	0.010
181	−13.273	**0.972**	0.018	0.024

^a^ Posterior probability ≥ 0.95 of negative selection; ^b^ posterior probability ≥ 0.95 of positive selection; ^c^ empirical Bayes factor for positive selection. Bold font represents the probabilities are above 0.95 or meaningful.

## Data Availability

Not applicable.
